# Paeoniflorin regulates macrophage activation in dimethylnitrosamine-induced liver fibrosis in rats

**DOI:** 10.1186/1472-6882-12-254

**Published:** 2012-12-13

**Authors:** Xiaorong Chen, Cheng Liu, Yunfei Lu, Zongguo Yang, Zhen Lv, Qingnian Xu, Qi Pan, Lingqing Lu

**Affiliations:** 1 Department of Traditional Chinese Medicine, Shanghai Public Health Clinical Center, 2901 Caolang Road, Jin Shan District, Shanghai, 201508, China; 2 Key Laboratory of Infectious Diseases of State Administration of Traditional Chinese Medicine (clinical base), Shanghai, 201508, China; 3 Scientific Research Center, Shanghai Public Health Clinical Center, Shanghai, 201508, China

**Keywords:** Liver fibrosis, Macrophage, Paeoniflorin

## Abstract

**Background:**

Macrophages in other organs (e.g. kidneys, lungs, and spleen, et. al) have rarely been reported in the development of liver fibrosis. Therefore, it is important to investigate macrophage activation in the main organs in liver fibrosis. We investigated the potential antifibrogenic effects of paeoniflorin (PF) in a dimethylnitrosamine (DMN)-induced rat model with special focus on inhibiting macrophage activation in the main organs.

**Methods:**

Rat hepatic fibrosis was induced by treatment with DMN three times weekly over a 4-week period. DMN rats were treated with water, PF, or gadolinium chloride (GdCl_3_) from the beginning of the 3^rd^ week. The expression of CD68, marker of macrophage, was investigated using immunohistochemical, real-time PCR, and western blot analysis.

**Results:**

Hepatic hydroxyproline content markedly decreased and histopathology improved in the DMN-PF rats. Expression of desmin and collagen 1 decreased notably in DMN-PF liver. CD68 expression in the liver, spleen and kidney increased markedly after 2 weeks but decreased in DMN-water rats. PF and GdCl_3_ decreased CD68 expression in the liver and spleen and there was no effect on kidney. CD68 expression in the lung increased gradually during the course of DMN-induced liver fibrosis, and PF inhibited CD68 expression in the lung significantly while GdCl_3_ increased CD68 markedly. Expression of tumor necrosis factor (TNF-α) was decreased significantly by GdCl_3_ in the liver, as revealed by real-time PCR analysis. However, GdCl_3_ could not decrease TNF-α level in the serum by enzyme linked immunosorbent assay (ELISA).

**Conclusions:**

Macrophage activation was disrupted in the liver, spleen, lung and kidney during development of DMN-induced liver fibrosis. PF administration attenuated DMN-induced liver fibrosis at least in part by regulating macrophage disruption in the main organs.

## Background

Hepatic fibrosis, which is characterized by progressive inflammation and deposition of extracellular matrix (ECM) components, is a common response to chronic liver disease [1]. Hepatic stellate cell (HSC) activation represents a critical event in fibrosis because these cells become the primary source of ECM in liver upon injury [2,3]. The macrophages can stimulate HSC activation by secreting tumor necrosis factor (TNF)-α [2]. To elucidate the role of macrophages in the pathogenesis of liver fibrosis, specific macrophage blocking agents such as gadolinium (III) chloride (GdCl_3_) have been used. When administered intravenously, GdCl_3_ inhibits phagocytosis by rat liver macrophages and selectively eliminates the large macrophages in the periportal zone of the acinus [3]. Accumulating evidence has suggested that macrophages play a key role in liver fibrosis [4-6], whereas macrophages in other organs (e.g. kidneys, lungs, and spleen) have been rarely reported and often neglected by those investigating development of liver fibrosis. In fact, other organs such as the spleen, lungs, and kidneys are all involved in liver fibrosis [7-9]. Thus, there is an urgent need to study the dynamic changes of macrophage activation in the main organs in liver fibrosis *in vivo*.

Paeoniflorin (PF), a monoterpene glycoside, is the principal bioactive component of *Radix Paeoniae Rubra* of Chinese traditional herbs. PF may be effective for treatment of stroke, as demonstrated using a rat middle cerebral artery occlusion model [10]. PF has been shown to attenuate the contraction induced by veratrine in isolated atria and aorta of rats [11]. It can inhibit nuclear factor κB activity of SGC-7901 cells, and enhance 5-fluorouracil-induced apoptosis of gastric carcinoma cells in a time- and dose-dependent manner [12,13]. PF might alleviate hepatic fibrosis caused by *Schistosoma Japonicum,* and the inhibitory effect might be associated with its ability to interfere with the interleukin (IL)-13 signaling molecule [14]. However, little is known about the effects of PF on macrophages in the main organs during the development of liver fibrosis.

To investigate the mechanism by which PF acts on liver injury, we investigated the potential antifibrogenic effects of PF in an experimental liver fibrosis rat model, with a special focus on the mechanisms regulating macrophage activation in the liver, spleen, kidneys and lungs. We were interested in: (1) whether macrophages in other organs such as the spleen, lungs and kidneys changed during dimethylnitrosamine (DMN)-induced liver fibrosis; and (2) the regulatory effects of PF on macrophages in other organs in DMN-induced liver fibrosis.

## Methods

### Main reagents

GdCl_3_ and PF were obtained from Sigma-Aldrich (St Louis, MO, USA), DMN was from Wako (Osaka, Japan). Sirius Red was obtained from Polysciences (St Louis, MO, USA) and dissolved in saturated picric acid (Chroma, Munster, Germany). SuperSignal West Pico Chemiluminescent Substrate was from Thermo Scientific (Rockford, IL, USA), SYBR Green Supermix was from Fermentas (Glen Burnie, MD, USA). Prestained protein marker was purchased from New England Biolabs (Beijing, China). Mouse desmin antibody was purchased from Abcam (Cambridge, UK) and diluted at a 1:100 ratio (ab8470), and mouse α-smooth muscle actin (SMA) mouse antibody was from Sigma and diluted at 1:400. Mouse glyceraldehyde 3-phosphate dehydrogenase (GAPDH) antibody was purchased from Kangchen (Shanghai, China) and diluted 1:5000, and mouse CD68 antibody was purchased from Serotec (Boston, MA, USA) and prepared at 1:100 dilution.

### Animals

Male Wistar rats were supplied by the Central Animal Care Facility of Shanghai Public Health Clinical Center (Permission No: SCXK 2007–0006). The rats received humane care with unlimited accesses to chow food and water during the study. All of the study protocols complied with the current ethical considerations of Shanghai Public Health Clinical Center’s Animal Ethic Committee and the procedural and ethical guidelines of the Chinese Animal Protection Act, which is in accordance with the National Research Council criteria. All animal experiments and procedures were reviewed and approved by the Institutional Animal Care and Use Committee (IACUC) of Shanghai Public Health Clinical Center and were performed in accordance with the relevant guidelines and regulations.

### Experimental design

Liver fibrosis was induced in adult rats over a 4-week period by three times weekly intraperitoneal injection of 10 mg/kg DMN dissolved in sterile saline [15]. At the end of the 2^nd^ week, three rats each from the control and DMN-treated groups were sacrificed to assess fibrosis development. At the beginning of week 3, DMN-treated rats were randomly assigned into the DMN-PF (n=7), DMN-GdCl_3_ (n=7), and DMN-water groups (n=7) for 2 weeks. Every group continued to receive weekly DMN treatment for a further 2 weeks, in addition to administration of PF, GdCl_3_, or water. The PF (20 mg/kg/day) was dissolved in water and administered by gavages. The GdCl_3_ in sterile saline was administered intravenously (i.v.) twice weekly without anesthesia through the tail vein at a dose of 7 mg/kg body weight [16]. At the end of the week 4, all of the animals were sacrificed. For sampling, excised livers were transferred to PBS solution, in which they were cut into pieces. These were either immediately shock frozen in liquid nitrogen and subsequently kept for further analyses or directly transferred into buffered 10% formaldehyde solution for paraffin embedding.

### Hydroxyproline assay

Snap-frozen liver samples (80–100 mg) were weighed, hydrolyze in 2.5 ml of 6M HCl at 110°C for 18 h [17] and h hydroxyproline content was expressed as μg/g liver.

### Liver function tests

Serum alanine aminotransferase (ALT), aspartate aminotransferase (AST), albumin (Alb) and total bilirubin (TBil) were evaluated in samples obtained at the end of the experiment. Activity was evaluated by using a commercial clinical test kit (Jiancheng Institute of Biotechnology, Nanjing, China) according to manufacturer’s instructions.

### Histopathology

The main organs such as liver, lungs spleen, kidneys, heart and brain were fixed in 4% paraformaldehyde, embedded in paraffin, and sectioned. Sections were stained with hematoxylin and eosin (H&E), Sirius red and immunostaining. Liver specimens were preserved in 10% formaldehyde, dehydrated in a graded alcohol series, embedded in paraffin blocks, sectioned to 5-μm thickness, placed on glass slides, and stained with Sirius red. Fibrosis was graded according to the method of Scheuer as follows: grade 0, normal liver; grade 1, increased collagen without formation of septa (small satellite expansion of portal fields); grade 2, formation of incomplete non-interconnecting septa, from portal tract to central vein; grade 3, complete but thin interconnecting septa, which divide the parenchyma into separate fragments; and grade 4, complete cirrhosis, similar to grade 3 but with thicker septa [18]. Three pathologists who were blind to the treatment of the rat performed pathological examinations. Fibrosis scores were given after thorough examination of three different areas of the tissue slides from each rat.

### Western blotting

Western blotting was performed according to standard methods, as previously described [19]. Tissue samples were homogenized in ice-cold RIPA buffer. Samples were then centrifuged for 10 min at 12,000 rpm. The supernatant was collected, and the protein concentration was measured using a commercial kit (Jiancheng Institute of Biotechnology, Nanjing, China). Protein lysates (50 μg) were separated by SDS-PAGE and subsequently transferred to nitrocellulose membranes. Membranes were blocked with 5% nonfat dry milk incubation buffer (BD, Sparks MD, USA) and incubated with antibodies against rat CD68 and α-SMA. Secondary antibody was used for chemiluminescent detection. Loading accuracy was evaluated by monoclonal antibodies against GAPDH.

### CD68 staining

After deparaffinization and dehydration, microwave antigen retrieval was performed for 5 min prior to peroxidase quenching with 3% H_2_O_2_ in PBS for 15 min. Subsequently, sections were preblocked with 5% bovine serum albumin for 30 min and incubated with a primary antibody (anti-CD68) overnight at 4°C. A negative control was treated as for the other samples except that primary antibodies were replaced with PBS and no staining took place. After washing in PBS, sections were incubated with biotinylated secondary antibody for 30 min and then stained with 3,3’-diaminobenzidine (Vector Laboratories, Burlingame, CA, USA) for 2–5 min. Slides were finally counterstained with hematoxylin for 2–3 min, mounted, and examined.

### RNA preparation, reverse transcription, and real-time quantitative PCR

Total RNA was isolated from liver tissue using Trizol Reagent (Invitrogen, Carlsbad, CA, USA) according to the manufacturer’s instructions. Two micrograms of RNA was reverse-transcribed to cDNA using M-MLV Transcriptase (Invitrogen, Carlsbad, CA, USA) in the presence of oligo-dT primers. Quantitative PCR was performed using SYBR Green I (Glen Burnie, MD, USA) for 40 cycles at 15 seconds at 95°C and 60 seconds at 60°C with Rotor-Gene 6000 (Corbett Research, Sydney, NSM, Australia) according to the manufacturer’s instructions. qPCR primers were are shown in Additional file 1: Table S1. Results were analyzed by double ΔCt method. Values were expressed as fold change in comparison with control.

### ELISA

The production of IL-1β (R&D Systems, Minneapolis, MN, USA) and TNF-α (Biosource, Carlsbad, CA, USA) in the serum were quantified using ELISA. ELISA was performed using the Quantikine Kit according to the manufacturer’s protocols.

### Statistical analysis

**Figure 1:**
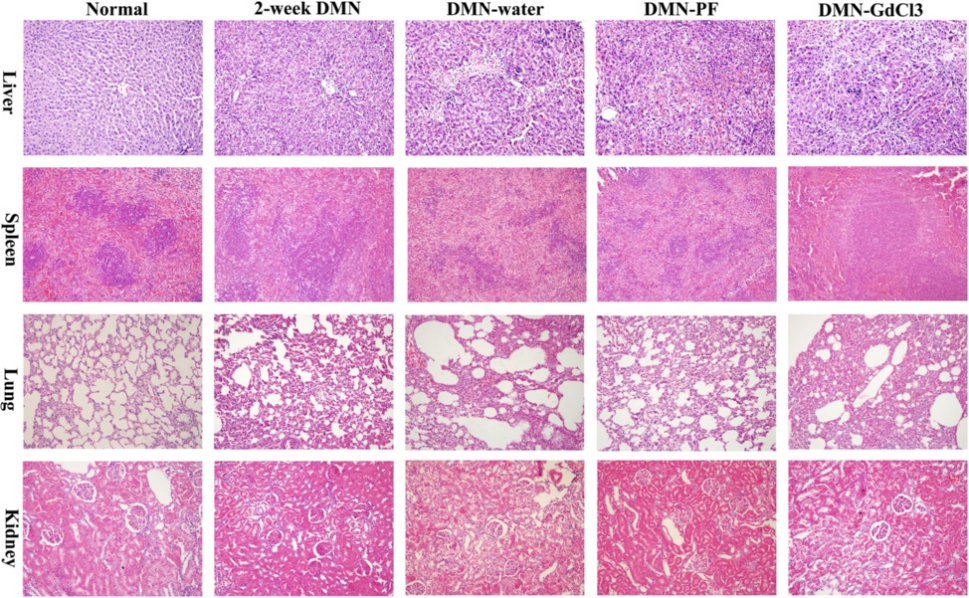
Effects of PF on H&E staining in different organs in DMN-induced rat liver fibrosis ×200 magnification.

**Figure 2:**
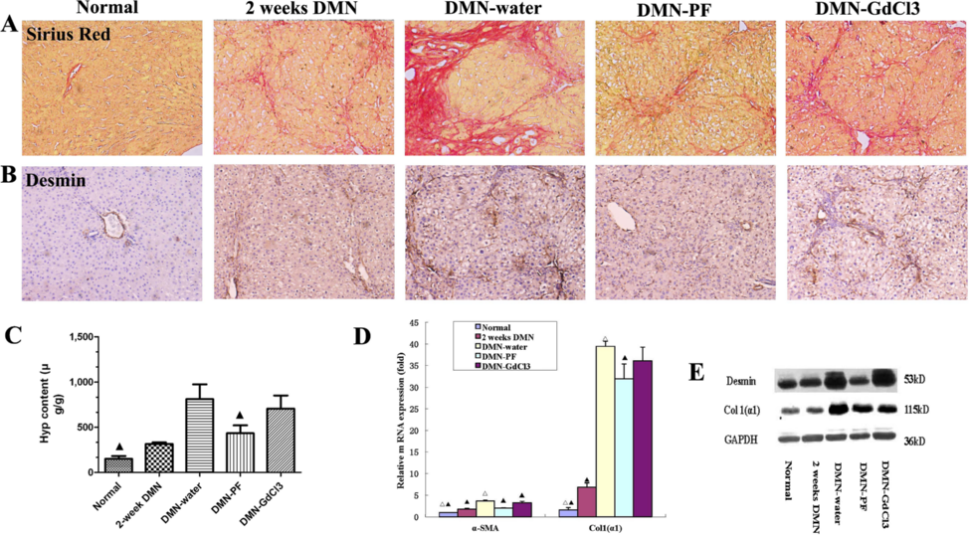
**Effects of PF on histological changes.A**, Sirius Red staining, ×100 magnification; **B**, desmin staining, brown staining indicates immunopositivity ×200 magnification, n=3; **C**, Hyp content; The number in Hyp and sirius red detection was the same as the number of animals in each group, data as mean ±SD, ^☆^*p*<0.05 versus 2-week DMN rats; ^⋆^*p*<0.05 versus DMN-water rats. Kruskal-Wallis test was used for group comparisons in Hyp. **D**, α-SMA and Col1(α1) using real-time PCR analysis, as an internal control, 18S rRNA was amplified (n=3), data as mean ±SD, ▴*p*<0.05 versus DMN-water rats; ^Δ^*p*<0.05 versus 2-week DMN rats. Bonferroni’s multiple comparison test were used for group comparisons in α-SMA and Col1(α1). **E**, the expression of desmin and Col1(α1) was analyzed using western blot in rat livers with DMN-induced liver fibrosis (n=3); western blotting of total protein extracts with antibodies recognizing desmin, Col1(α1), and GAPDH respectively.

All the results are expressed as mean ± standard deviation (SD). For group comparisons, if the samples satisfied normality assumption, a one-way analysis of variance Bonferroni’s multiple comparison test was applied; If the samples failed normality assumption, the non-parametric Kruskal-Wallis statistic Dunn’s multiple comparison test. The analyses were performed with GraphPad Prism 5.0 (GraphPad Software, Inc., La Jolla, CA). Rank data (Additional file 1: Table S3) were analyzed with ridit. Other data used in the manuscript are all belong to measurement data such as Tables 1, 2, 3, and 4, Figure 1C/ 1D, Figure 2A, and Additional file 1: Table S2, et al. A *P* value < 0.05 was considered statistically significant.

**Table 1:** **Effects of PF on body weight and main organs weight in DMN-induced rat liver fibrosis** ( $\bar{x}\pm s$**)**

**Group (number)**	**Body weight (g)**	**Liver weight (g)**	**Spleen weight (g)**	**Heart weight (g)**	**Lung weight (g)**	**Kidney weight (g)**	**Brain weight (g)**
2-week normal(3)	199.33±9.45	6.81±0.61	0.54±0.11	0.56±0.09	0.75±0.12	1.16±0.10	1.04±0.02
2-week DMN(3)	180.33±0.55	7.67±0.47	0.77±0.06^⋆^	0.50±0.01^▴^	0.93±0.06^⋆^	1.37±0.06	1.10±0.00
4-week normal(7)	273.90±12.29▴	10.03±0.86▴	0.59±0.09^⋆^	0.81±0.07^▴^	1.00±0.00	1.64±0.15^▴^	1.17±0.05
DMN-water(5)	201.50±17.81	5.92±1.64	1.38±0.30^☆^	0.72±0.07^△^	1.05±0.11^☆^	1.43±0.08	1.20±0.10
DMN-PF(7)	208.87±18.46	6.45±1.21	1.13±0.22	0.66±0.05	1.00±0.00	1.46±0.13	1.17±0.05
DMN-GdCl_3_(6)	183.48±13.99^▴^	4.73±0.41	1.11±0.08	0.63±0.08^▴^	1.07±0.10	1.65±0.26^▴^	1.13±0.12

^△^*p*<0.05 versus the same period in 2-week DMN rats; ^▴^*p*<0.05 versus the same period in DMN-water rats.

Bonferroni’s multiple comparison test were used for group comparisons in body weight, liver weight, heart weight, and kidneys weight.

^☆^*p*<0.05 versus the same period in 2-week DMN rats; ^⋆^*p*<0.05 versus the same period in DMN-water rats. Kruskal-Wallis test were used for group comparisons in liver weight, spleen weight, lung weights, and brain weight.

**Table 2:** **Effects of PF on serum parameters in DMN-induced rat liver fibrosis** ( $\bar{x}\pm s$**)**

**Group(number)**	**ALT(U/L)**	**AST(U/L)**	**ALP(U/L)**	**TP (g/L)**	**Alb (g/L)**	**TBil(μmol/L)**
Normal (10)	25.14±2.86^△^▴	79.00±5.83^△^▴	226.71±24.68^△^▴	57.60±1.95^△^▴	29.15±1.13^△^ ▴▴	2.40±0.08^⋆^
2-week DMN (3)	90.33±3.21▴	182.67±11.93▴	395.00±53.11▴	48.53±4.55▴	26.33±1.82▴▴	3.30±0.17^⋆^
DMN-water (5)	256.80±144.26^△^	549.40±260.71^△^	667.60±41.20^△^	30.82±5.02^△^	18.94±0.89^△△^	79.00±21.13^☆^
DMN-PF (7)	127.43±20.19▴	234.86±42.56▴	394.00±34.03▴	46.98±3.73▴	25.37±1.96^▴▴^	31.97±12.34^⋆^
DMN-GdCl_3_(6)	230.20±43.27	425.60±79.67	610.80±51.16▴	34.14±3.06	20.68±3.05	68.96±15.07

^Δ^*p*<0.05 versus 2-week DMN rats; ▴*p*<0.05 versus DMN-water rats. Bonferron’s multiple comparison test were used for group comparisons in ALT, AST, ALP, TP, and Alb.

^☆^*p*<0.05 versus 2-week DMN rats; ^⋆^*p*<0.05 versus DMN-water rats. Kruskal-Willam test were used for group comparisons in TBil

**Table 3:** **Effect of PF on TNF-α and IL-1β levels in the serum in DMN-induced rat liver fibrosis** ( $\bar{x}\pm s$**)**

**Group (number)**	**TNF-α (pg/L)**	**IL-1β (pg/L)**
Normal (5)	35.44±5.64^△^▴	26.70±3.56^△^▴
2-week DMN (3)	896.51±78.06▴	954.71±75.28▴
DMN-water (5)	758.72±61.54^△^	647.66±95.54^△^
DMN-PF (5)	446.95±42.90▴	471.34±26.67▴
DMN-GdCl_3_ (5)	708.03±78.84	705.63±99.46

^△^*p*<0.05 versus 2-week DMN rats; ^▴^*p*<0.05 versus DMN-water rats. Bonferroni’s multiple comparison test was used for group comparisons in TNF-α and IL-1β.

**Table 4:** **Effects of PF on transcript levels of proinflammatory cytokines TNF-α and IL-1β in different organs in DMN-induced rat liver fibrosis** ( $\bar{x}\pm s$**)**

**Group (number)**	**Liver**		**Spleen**		**Lung**		**Kidney**	
	**IL-1β (n-fold)**	**TNF-α (n-fold)**	**IL-1β (n-fold)**	**TNF-α (n-fold)**	**IL-1β (n-fold)**	**TNF-α (n-fold)**	**IL-1β (n-fold)**	**TNF-α (n-fold)**
Normal (3)	1.20±0.18^△△^▴▴	1.30±0.29^△^▴	1.38±0.40^△^▴	1.41±0.50^△^▴	1.14±0.27^△^▴	1.44±0.47^△^▴	1.36±0.37^△^	1.33±0.28^△^
2-week DMN (3)	4.98±0.67	11.64±2.24▴	4.74±1.49	3.71±0.44	9.00±1.39	7.41±1.37▴	4.46±0.68▴	6.72±1.68▴
DMN-water (3)	3.89±0.72	6.09±1.86^△^	3.80±1.36	3.38±0.47	11.09±3.34	11.24±2.13^△^	1.99±0.53^△^	2.24±0.57^△^
DMN-PF (3)	2.97±0.39	4.50±0.74	2.82±0.30	2.28±0.34▴	4.09±0.97▴	3.13±0.28▴	1.76±0.19	1.70±0.19
DMN-GdCl_3_ (3)	3.52±0.84	3.49±0.75▴	3.37±1.16	2.68±0.46	9.73±1.85	10.07±2.53	1.69±0.30	1.55±0.45

^Δ^*p*<0.05 versus 2-week DMN rats; ▴ *p*<0.05 versus DMN-water rats. Bonferroni’s multiple comparison test were used for group comparisons.

## Results

### PF improved survival rates in DMN-indcued liver fibrosis in rats

During drug intervention, 2 rats died in the DMN-water group, and 1 in the DMN-GdCl_3_ group. Animal body, liver, lung, spleen, kidney, heart and brain weights were monitored during the formation of liver fibrosis (Table 1). Liver, spleen and lung weights increased significantly in the 2-week DMN rats compared with those in the 2-week normal group (*p*<0.05). Compared with 4-week normal rats, body, liver, heart and kidney weights decreased significantly in the 4-week DMN-water group (*p*<0.05), whereas the spleen weight in the 4-week DMN-water rats increased significantly (*p*<0.05). These results indicated that other organs such as spleen, lungs, kidneys and heart were damaged severely during cirrhosis. Compared with 4-week DMN-water rats, body, spleen and heart weights decreased notably in the DMN-GdCl_3_ rats (*p*<0.05), and spleen weight decreased significantly in the DMN-PF rats (*p*<0.05).

Liver/body weight ratio of the DMN-water group was significantly lower than that of the normal group (*p*<0.05). Spleen/body, lung/body, kidney/body, heart/body and brain/weight ratios were markedly increased (*p*<0.05) in the 4-week DMN-water rats compared with those in the 4-week normal rats (Additional file 1: Table S2). The liver/body weight ratio of the PF rats was higher than that of the DMN-water group but there were no significant difference (*p*<0.05).

### PF ameliorated liver function in DMN-induced liver fibrosis

Liver function parameters deteriorated over time in rats subjected to DMN (Table 2). DMN-treated rats developed hepatic injury as shown by significantly higher plasma concentrations of AST, ALT, ALP and TBil, and a lower concentration of TP and Alb compared with normal rats. PF treatment ameliorated significantly the increase of ALT, AST, ALP and TBil, and the decrease of Alb compared with 4-week DMN-water rats (*p*<0.05), while GdCl_3_ decreased ALP levels significantly (*p*<0.05).

### PF improved histology in DMN rats

Pathological changes occurred in the liver during development of fibrosis, which was confirmed by H&E staining, and in the spleen, lungs and kidneys (Figure 1). In the liver, there was normal lobular architecture, with the central vein and radiating hepatic cords in the livers of normal rats (Figure 1). After DMN intoxication for 2 weeks, we observed massive hepatocyte necrosis, intense neutrophil infiltration, and initiation of fibrosis (Figure 1). In the DMN-water group, the liver sections revealed collagen fiber deposition, marked cirrhosis, and severe centrilobular necrosis. We observed marked reduction in the thickening of the collagen bundles in the DMN-PF group.

In normal spleen, the white pulp was circular or oval and consisted mainly of lymphocytes and the red pulp contained mainly red blood cells, and macrophages surrounded the white pulp (Figure 1). The spleen showed clear structural damage in the 2-week DMN rats; the white pulp was irregular in shape and the circular region had almost disappeared. The white pulp structure was restored in the 4-week DMN rats. GdCl_3_ had a positive protective effect on spleen structural restoration.

In normal lungs, there was no inflammatory cell infiltration and alveolar structure was normal. Alveolar structural damaged gradually developed after DMN treatment (Figure 1). There was a large amount of inflammatory cell infiltration in the alveolar walls and spaces. PF could reduce alveolar inflammatory cell invasion significantly in the lungs.

Normal kidney was characterized by clear organizational structure, and there was almost no macrophage infiltration (Figure 1). A lot of renal tubular epithelial cell swelling, necrosis and inflammatory cell infiltration appeared in 2-week DMN rats. Macrophage infiltration clearly decreased in kidneys of 4-week DMN rats. However, there was no obvious effect on the brain and heart compared with other organs in DMN-induced liver fibrosis (data not shown).

### PF inhibited liver fibrosis in DMN rats

We evaluated whether PF inhibited hepatic fibrosis. Sirius Red staining clearly revealed accumulation of ECM, especially collagen. As shown in Figure 2A, collagen staining was scarcely observed in the normal liver samples, except in the area around the small central venous walls. In DMN-treated liver samples at 2 weeks, collagen stretched from the portal to lobular areas (Figure 2A). Additional cirrhotic nodule formation was seen in 4-week DMN-treated rats. Liver fibrosis was attenuated extensively by PF, which had better effects than GdCl_3_. Ridit analysis showed that there was a significant difference between the DMN-PF and DMN-water rats (*p*<0.05), but the DMN-GdCl_3_ intervention group did not differ significantly (Additional file 1: Table S3).

Using antibody against desmin, a marker of stellate cell activation, we assayed the expression of this protein immunohistochemically (Figure 2B). In the normal rats, only vascular smooth muscle cells were strongly positive for desmin. After 2 weeks DMN treatment, desmin-positive HSCs were detected in areas of centrilobular and near periportal fibrotic bands. The number of desmin-positive HSCs was even higher in 4-week DMN-treated cirrhotic livers. After treatment with PF, the increased expression of desmin was markedly decreased. These results revealed the antifibrogenic effects of PF. Hepatic hydroxyproline content gradually increased in DMN-treated rats gradually (Figure 2C). PF treatment ameliorated significantly the increase of hydroxyproline content, compared with 4-week DMN-water rats (*p*<0.05).

Sustained deposition of ECM is mainly resulted from activation of HSCs; therefore, a correlation between accumulated collagen and activated HSCs was studied by detecting a marker of activated HSCs, α-SMA in liver sections described above. We detected the α-SMA and Collagen 1 α1 (Col1α1) by real-time PCR (Figure 2D). There was a gradual increase in their expressions and PF significantly attenuated the upregulation of α-SMA and Col1 (α1) significantly. Changes in expression of desmin and Col1 (α1) were also demonstrated by western blotting (Figure 2E).

Taken together, these results confirm that DMN administrations cause HSC activation and accumulation of ECM that might facilitate liver fibrosis and PF could inhibit DMN-induced liver fibrosis.

### Macrophage activation disturbed in main organs in DMN-induced liver fibrosis

**Figure 3:**
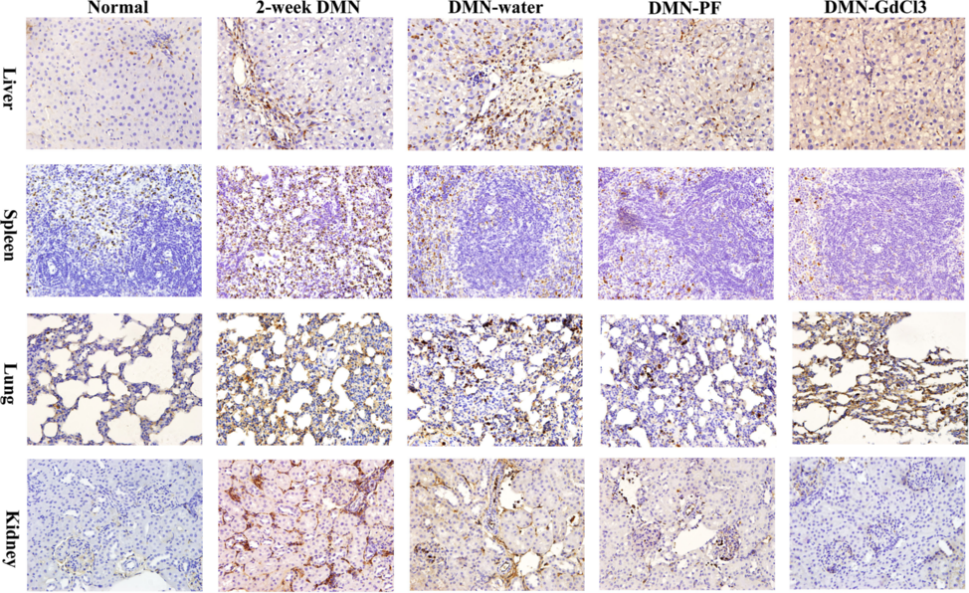
**Effects of PF on CD68 using immunohistological staining in different organs in DMN-induced rat liver fibrosis.** CD68 was investigated by brown staining indicates immunopositivity ×400 magnification, n=3.

Liver macrophages/Kupffer cells were important contributors to fibrosis progression. However, macrophages in other organs have rarely been detected in previous studies. To elucidate the function of macrophages in liver fibrosis, a specific macrophage marker, CD68, has been used to monitor macrophage activation [4]. As shown in Figure 3, CD68-positive macrophages were present in hepatic sinusoids and were at very low levels in normal liver. After DMN treatment, CD68-positive macrophages with strong staining appeared not only in hepatic sinusoids, but also in portal areas and adjacent to fibrotic septa. PF and GdCl_3_ decreased CD68 expression compared with that in DMN-water rats.

The CD68-positive macrophages were at a relative high level and mainly located in the red pulp in normal spleen (Figure 3). CD68-positive macrophages increased significantly around the disrupted white pulp in 2-week DMN rats and decreased markedly in DMN-water rats. PF and GdCl_3_ inhibited CD68-positive macrophages markedly.

CD68-positive pulmonary macrophages with low expression in the lung in normal rat distributed widely in the alveolar, bronchial, pulmonary interstitial. Macrophage distribution increased gradually following DMN treatment and reached a peak in 4-week DMN-water rats. PF decreased the number of CD68-positive macrophages significantly; however, GdCl_3_ had no such effect.

Immunostaining with CD68 antibody showed marked accumulation of CD68-positive macrophages in the kidneys after 2 weeks DMN treatment. However, CD68-positive macrophages in the kidneys were decreased markedly in DMN-water rats. Administration of PF and GdCl_3_ had no obvious effects on macrophages in kidneys compared with that in DMN-water rats.

**Figure 4:**
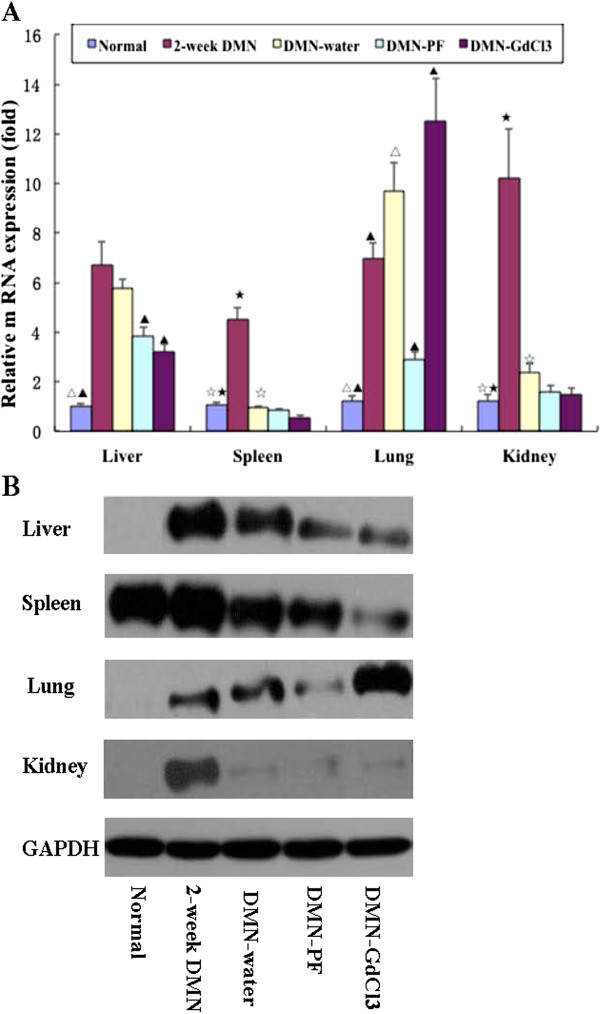
**Effects of PF on CD68 expression in different organs in DMN-induced rat liver fibrosis.** (**A**) CD68 was measured by real-time PCR. As an internal control, 18S rRNA was amplified. The data represented the mean ± SD. ^▴^*p*<0.05 and ^▴▴^*p*<0.01 versus DMN-water rats; ^△^*p*<0.05 and ^△△^*p*<0.01 versus 2-week DMN rats; LSD test were used for group comparisons in CD68 detection in the liver and lung. *p*<0.05 versus 2-week DMN rats; ^⋆^*p*<0.05 versus DMN-water rats Kruskal-Wallis test were used for group comparisons in CD68 detection in the spleen and kidney, n=3. (**B**) CD68 were detecting by western blotting.

Real-time PCR showed that CD68 transcripts were upregulated about 6.7-fold after 2 weeks DMN treatment (Figure 4A). Thereafter, mRNA expression declined slightly in the 4-week DMN-treated liver. Similar changes at the protein level of CD68 were observed by western blotting (Figure 4B). Expression of CD68 in the spleen and kidneys was similar. CD68 expression in the spleen and kidneys in DMN-water rats decreased to a level below that in normal rats. PF and GdCl_3_ did not inhibit CD68 expression significantly in the spleen and kidneys. Unlike CD68 expression in the liver, kidneys and spleen, it increased gradually following DMN administration in the lungs. PF reduced CD68 expression, whereas GdCl_3_ even increased CD68 expression. These results were also confirmed by western blotting.

### PF but not GdCl_3_ inhibited serum levels of TNF-α and IL-1β

One of important function of macrophages is to secrete proinflammatory factors, so we decided to detect proinflammatory cytokines in the serum. The level of TNF-α and IL-1β was low in normal serum and increased to 25.3- and 35.7-fold following 2 weeks DMN-treatment, respectively (Table 3), compared with that of the normal rats, and decreased markedly in DMN-water rats. PF but not GdCl_3_ decreased TNF-α and IL-1β significantly. The cytokines content in the serum represents the overall level in the body, and these proinflammatory cytokines are mainly derived from macrophages.

### Inflammatory cytokines could derive from other organs besides the liver

We detected TNF-α and IL-1β in the liver, spleen, lungs and kidneys using real-time PCR. IL-1β transcripts were up-regulated 4.15-fold, 3.43-fold, 7.89-fold and 3.28-fold in the liver, spleen, lungs, and kidneys, respectively, in 2-week DMN rats (Table 4). Afterwards, IL-1β decreased in the liver, spleen and kidneys in DMN-water rats while it increased in the lungs in DMN-water rats (*p*>0.05) compared with that in the 2-week DMN rats. PF could inhibit the IL-1β transcription significantly in the lungs compared with DMN-water rats (Table 4).

GdCl_3_ treatment decreased liver TNF-α transcripts markedly compared with that of DMN-water rats. However, GdCl_3_ almost did not decrease TNF-α transcripts in the lungs. PF significantly suppressed the TNF-α expression in the spleen and lungs significantly (p <0.01 or 0.05) compared with that of DMN-water rats (Table 4).

## Discussion

The present study demonstrates that macrophage activation is disrupted in the liver, spleen, lungs and kidneys during liver fibrosis development. The macrophages in the lungs might also play a key role in DMN-induced liver fibrosis. PF administration attenuates liver fibrosis at least in part by regulating the macrophages in the main organs.

Liver fibrosis is considered as an outcome of chronic liver injury during long-term wound healing, which causes increasing amounts of ECM deposition in the liver and eventually leads to liver fibrosis and later cirrhosis [20-22]. Despite advances in the understanding of liver fibrosis, the landmark isolation of HSCs and demonstration of their *in vitro* activation resulted in 30 years of fibrosis research focused on understanding HSC behavior in culture and applying these findings to animal models of disease [23]. However, translation of this knowledge into antifibrotic therapies has a long way to go. All these results have indicated that microenvironment and the “whole conception” are more important than just focusing only on one cell type.

Inflammation to repeated injury and repair is at the root of hepatic fibrosis [24]. The inflammatory process that results from injury is characterized by the production of soluble mediators, including cytokines such as TNF-α and IL-1β derived from macrophages. Prior clinical and experimental evidence indicates that the inflammatory response in cirrhosis is not restricted to the liver, but extends to the systemic circulation and other organs such as the spleen, lungs, kidneys, et al. [25,26]. Given the pathogenic role of inflammation in tissue damage in cirrhosis, modulation of the inflammatory response is a therapeutic target. Researchers have reported that simultaneous administration of GdCl_3_ prevents pig serum and DMN-induced rat liver fibrosis with reduced the expression of α-SMA and procollagen I [27]. Ide reported that pretreatment with GdCl_3_ had less prominent therapeutic effects in thioacetamide-induced liver fibrosis [28]. There was a problem in all these studies, in that researchers detected the macrophage function in liver fibrosis using specific blocker GdCl_3_, while macrophages in other organs were always neglected and rarely reported. What about the proinflammatory cytokines in other organs in liver fibrosis? These soluble proinflammatory cytokines within the blood reached the liver, thereby affecting the process of liver fibrosis. Therefore, urgent investigation is needed of these cytokines in other organs in liver fibrosis.

Liver injury could be associated with macrophage activation and trigger migration of macrophages into hepatic cords in which they stimulate fibrosis by secreting proinflammatory cytokines. In fact, liver fibrosis is a systemic disease that involves many organs such as the spleen, kidneys, and lungs. The current results showed that macrophages and proinflammatory cytokines were disrupted in the liver, lungs, spleen, and kidneys during the development of liver fibrosis. Other researchers have shown that macrophage activation in the brain in hepatitis-C-virus-infected patients [29]. However, we did not detect CD68 expression in the brain and heart during DMN administration, which may have been due to expression of CD68 being too low to detect using western blotting.

We showed that macrophages in different organs varied widely and GdCl_3_ had different effects on them. The trends in expression of CD68 and proinflammatory cytokines in the spleen and kidneys were similar in some extent; they reached a peak in 2-week DMN rats, and then decreased significantly in 4-week DMN rats. These results indicated that proinflammatory cytokines might derive mainly from macrophages; and that macrophages in kidneys and spleen could play a lesser role, because they decreased to near or below the normal levels in 4-week DMN rats. However, the expression of CD68 and proinflammatory cytokines increased gradually in the lung following DMN-treatment, but to our surprise, GdCl_3_ did increase CD68 expression in the lungs compared with that in DMN-water rats. Based on our knowledge, GdCl_3_ definitely had an inhibitory effect on liver macrophages (Kupffer cells). However, the effects of GdCl_3_ on macrophages in the lungs are more controversial. Some researchers have reported that GdCl_3_ has no effect on lung macrophages (at the dose of 10 or 20 mg/kg, i.v.) [30,31], but others have demonstrated in increase in the number of macrophages and expression of inflammatory mediators after GdCl_3_ treatment (10 mg/kg, i.v.) [32]. There were two reasons for this phenomenon. First, signals from proinflammatory cytokines could enhance the expression of adhesion molecules and chemotactic factors, leading to increases in the number of infiltrating macrophages into the lungs [33]. TNF-α mRNA expression was increased after GdCl_3_ treatment in the lungs of rats with DMN-induced liver fibrosis. The TNF-α signal induced macrophage infiltration. Second, Kupffer cells have been suggested to be heterogeneous macrophage lineage cells and have two subpopulations (ED1-specific marker CD68, and ED2-specific marker CD163), based on their phenotype and function [34]. GdCl_3_ can inhibit/inactivate ED2 macrophages but does not inhibit ED1 macrophages, and it has been reported that ED2 macrophages can transform into ED1 macrophages [34]. GdCl_3_ might inhibit ED2 macrophages, which are transformed into ED1 macrophages in the lungs in DMN-induced liver fibrosis, so the number of ED1 macrophages (CD68 expression) increased in the lungs.

Although GdCl_3_ decreased IL-β and TNF-α in the liver, spleen and kidneys, it did not reduce proinflammatory cytokines significantly in the serum and lung tissue. These may be related to the GdCl_3_-induced increase in CD68 and proinflammatory cytokines, which leads to no significant inhibitory effects in the serum, and no marked improvement in liver fibrosis. PF could decrease CD68 expression significantly in the lungs and liver. PF decreases proinflammatory cytokine expression markedly in the lungs and serum. By comparison, the anti-fibrotic effects of GdCl_3_ and PF in DMN-induced liver fibrosis, we could conclude that macrophages in other organ, at least in part, play an important role in liver fibrosis.

## Conclusions

The present study demonstrated that PF is able to improve the histological changes and reduce ECM accumulation during DMN-induced liver fibrosis. The relevant mechanism involves inhibiting macrophage disruption in the liver and lungs. These results indicate that one single-targeted blocker is limiting and giving rise to a new concept, in which liver fibrosis is not restricted to the liver, but also involves many other organs. Macrophage activation is disrupted in the liver, spleen, lungs and kidneys in DMN-induced liver fibrosis development. PF administration attenuates DMN-induced liver fibrosis at least in part by regulating the disrupted macrophages in the main organs.

## Abbreviations

α-SMA: Smooth muscle actin alpha; Col 1(α1): Collagen 1alpha 1; DMN: Dimethylnitrosamine; ELISA: Enzyme linked immunosorbent assay; GAPDH: Glyceraldehyde-3-phosphate dehydrogenase; GdCl_3_: Gadolinium chloride; HSCs: Hepatic stellate cells; IL: Interleukin; KCs: Kupffer cells; PF: Paeoniflorin; PCR: Polymerase Chain Reaction; TNF-α: Tumor necrosis factor alpha.

## Competing interests

The authors declare that they have no competing interests.

## Authors’ contributions

XC and CL conceived of the study design. LZ, QP, LL and QX performed the data analysis. CL drafted the manuscript, ZY and ZL perform the experiments. All authors confirm that the content of this paper has not been published elsewhere and does not overlap or duplicate their published work. All authors have read and approved the final manuscript.

## Pre-publication history

The pre-publication history for this paper can be accessed here:

http://www.biomedcentral.com/1472-6882/12/254/prepub

## Supplementary Material

Additional file 1: Table S1 PCR primers used in this study. **Table S2.** Ratio of liver, spleen, heart, lung, kidney and brain weight/body weight in DMN-induced rat liver fibrosis (2-1). Ratio of liver, spleen, heart, lung, kidney and brain weight/body weight in DMN-induced liver fibrosis in rats (2-1). **Table S3.** Effects of PF on fibrotic grade and Hyp content in DMN-induced rat liver fibrosis. Click here for file
