# Supplementary material for: Paeoniflorin regulates macrophage activation in dimethylnitrosamine-induced liver fibrosis in rats
Source: BMC Complement Altern Med. 2012 Dec 13;12:254. doi: 10.1186/1472-6882-12-254 (PMC3575304; doi:10.1186/1472-6882-12-254)
Supplement: Additional file 1: Table S1 — PCR primers used in this study. Table S2. Ratio of liver, spleen, heart, lung, kidney and brain weight/body weight in DMN-induced rat liver fibrosis (2-1). Ratio of liver, spleen, heart, lung, kidney and brain weight/body weight in DMN-induced liver fibrosis in rats (2-1). Table S3. Effects of PF on fibrotic grade and Hyp content in DMN-induced rat liver fibrosis. [file 1472-6882-12-254-S1.pdf]

Supporting materials

Table 1 PCR primers used in this study

Gene	Forward primer	Reverse primer	Gen Bank Accession No.	Length (bp)
CD68	5'-TGTACCTGACCCAGGGTGGAA-3'	5'-GAATCCAAAGGTAAGCTGTCCGTAA-3'	NM_001031638	140
TNF-α	5'-GGCAGCCTTGTCCCTTGAAGAG-3'	5'-GTAGCCCACGTCGTAGCAAACC-3'	X6659	171
IL-1β	5'-GCTGTGGCAGCTACCTATGTCTTG-3'	5'-AGGTCGTCATCATCCCACGAG-3'	NM_031512	120
α-SMA	5'-CGAGAGGACGTTGTTAGCATAGAG-3'	5'-GGGCATCCACGAAACCA-3'	BI282702	85
COL-1(α)1	5'-ACTCAGCCGTCTGTGCCTCA-3'	5'-GGAGGCCTCGGTGGACATTA-3'	NM_007742	183
18SrRNA	5'-GTAACCCGTTGAACCCCATT-3'	5'-CCATCCAATCGGTAGTAGCG-3'	X0117	151

Table 2 Ratio of liver, spleen, heart, lung, kidney and brain weight/body weight in DMN-induced rat liver fibrosis (2-1).

Group (number)	liver/ body %	spleen/body ‰	heart/body‰
2-week normal（3）	3.42±0.29	2.70±0.49△	2.80±0.36
2-week DMN（3）	4.25±0.26	4.25±0.33▲	2.79±0.04▲
4-week normal（7）	3.66±0.15	2.15±0.37▲	2.97±0.18▲
DMN-water（5）	2.91±0.66	6.78±1.07△	3.59±0.48△
DMN-PF（7）	3.08±0.44	5.38±0.71▲	3.15±0.30▲
DMN-$GdCl_3$（6）	2.58±0.09	4.86±1.81▲	3.46±0.49

Ratio of liver, spleen, heart, lung, kidney and brain weight/body weight in DMN-induced liver fibrosis in rats (2-1)

group(number)	lung/body‰	kidney/body‰	brain/body‰
2 weeks normal（3）	3.76±0.55△	5.80±0.25	5.21±0.25
2weeks DMN（3）	5.18±0.31	7.58±0.30	6.10±0.02
4 weeks normal（7）	3.66±0.17▲	6.00±0.49	4.29±0.30★
DMN-water（5）	5.22±0.54	7.16±0.98	5.97±0.36
DMN-PF（7）	4.82±0.42	6.99±0.46	5.66±0.66
DMN-$GdCl_3$（6）	5.83±0.69▲	9.07±1.80	6.23±0.98

△p<0.05 versus the same period in 2-week DMN rats; ▲p<0.05 versus the same period in DMN-water. Bonferroni's multiple comparison tests were used for group comparisons in ratio of spleen/body, heart/body, lung/body.

☆p<0.05 versus the same period in 2-week DMN rats; ★p<0.05 versus the same period in

DMN-water rats. Kruskal-Wallis test were used for group comparisons in ratio of liver/body, kidney/body, and brain/body.

Table3 Effects of PF on fibrotic grade and Hyp content in DMN-induced rat liver fibrosis

Group	*n*	Hyp content ug/g($\bar{x} \pm s$)	Fibrotic stage					Ridit value
			0	I	II	III	IV	
Normal	10	150.36±30.40★	10	0	0	0	0	0.1613★
2-week DMN	3	315.43±17.24	0	0	2	1	0	0.4570
DMN-water	5	812.09±163.65	0	0	0	1	4	0.8161
DMN-PF	7	434.17±87.94★	0	0	2	5	0	0.5368★
DMN-$GdCl_3$	6	707.73±144.85	0	0	0	2	4	0.7796

Grades 0, normal; 1, very slight; 2, slight; 3, moderate; and 4, severe; data as numbers of animals with each fibrotic grade; ★$p<0.05$ vs DMN-water rats; ☆$p<0.05$ vs 2-week DMN rats. Kruskal-Wallis test were used for group comparisons in Hyp content and ridit analysis.
